# Remembering Dr. Rex Munday

**DOI:** 10.3390/toxins9090257

**Published:** 2017-08-24

**Authors:** Luis M. Botana, Vitor Vasconcelos

**Affiliations:** 1 Departamento de Farmacologia, Facultad de Veterinaria, Universidad de Santiago de Compostela, 27002 Lugo, Spain; 2 Interdisciplinary Center of Marine and Environmental Research—CIIMAR, Porto University, Terminal de Cruzeiros do Porto de Leixões, 4050-208 Matosinhos, Portugal; 3 Department of Biology, Faculty of Sciences, Porto University, 4069-007 Porto, Portugal

**Keywords:** Rex Munday, toxicology of natural products, free radical toxicology, cancer chemoprevention

## Abstract

Rex Munday was a scientist working for AgResearch Ltd. in New Zealand. He was a leading figure in the area of marine toxin toxicity. His passing in July 2017 marked a loss for his family, as well as for colleagues who knew him as a dedicated professional, and a lively scientist with a great sense of humor.

“It is very sad for me to write this note, since with the loss of Dr. Rex Munday, our struggle to understand the fantastic world of aquatic toxins has been greatly diminished. I met Rex for the first time 12 years ago in Cesenatico, Italy. At the time, I was organizing a toxicology meeting to define threshold levels for all marine toxins. It was a one day meeting and I asked Rex to join us so that he could bring not only his experience but also the collective view of the very active group of researchers from ‘down under’. In Cesenatico, I met a sweet person, with a very strong position with regard to what was to be done for the toxicology of marine toxins. In that meeting, he was a natural leader of the group, and his input helped the meeting to go fast and well. After that, I was able to convince him to join us in Cyprus for another meeting of the European Network of Reference Laboratories, and again, he came, and became the main voice and everyone listened to him. He attended both meetings, even though he was having trouble with the length of the trips, as it was difficult for him to be sitting for so long due to his cardiovascular issues. He was very passionate about doing the right toxicological studies for marine toxins, and actually my group is following his advice ever since the first time we talked. The review he wrote for my 3rd edition of the phycotoxins book is to me the best review ever written in the topic of toxicology of marine toxins. When he was required for a FAO meeting in Rome, two years ago, he was not able to attend due to his health condition, and shared his experience with us by phone.

I have met Rex very few times, and yet we were good friends. We were in frequent contact and to me his opinion guided many of our experiments. There is a picture, Figure 1, I have from the reception at the hotel in Cesenatico that I shared with him and Dr. T. Yasumoto, and it appears often in my computer screen saver. There, he smiles and looks at the camera with a blend of sweetness and sadness that always makes me think fondly of him when I see it. To me, Rex was an important person, and I will miss him.”

Rex Munday was a scientist working for AgResearch Ltd. in New Zealand. He was a leading figure in the area of toxicity of marine toxins. His passing in July 2017 marked a loss for his family, as well as for colleagues who knew him as a dedicated professional, and a lively scientist with a great sense of humor.

Rex had a diversified and rich career, lately dominated by studies on mechanisms of action and toxicity of many marine toxins produced by dinoflagellates and diatoms. In his first years of research, his studies were mostly associated with several human and animal diseases such as cancer and diabetes, using rats as model organisms, and unraveling mechanisms of toxicity. In 2002, he published the first paper on marine toxins reporting a palytoxin-like toxin from *Ostreopsis siamensis*. Since then, he produced multiple studies on the toxicity of pectenotoxins, gimnodimines, yessotoxins, domoic acid, maitotoxins, ciguatoxins, and pinnatoxins, to mention a few. He was a key person in the area of the toxicology of marine toxins, recently publishing seminal papers on the toxicity of spirolides, questioning the implications of protein phosphatases in the toxic effects of okadaic acid, and unraveling the toxicity of several poorly characterized saxitoxin analogues such as gonyautoxin 5, gonyautoxin 6, decarbamoyl gonyautoxin 2 and 3, decarbamoyl neosaxitoxin, C-1 and 2 and C-3 and 4.

Rex Munday will be missed by his many friends and colleagues in the marine toxins field, not only for his scientific excellence, but also for being a kind gentleman.

## Figures and Tables

**Figure 1:**
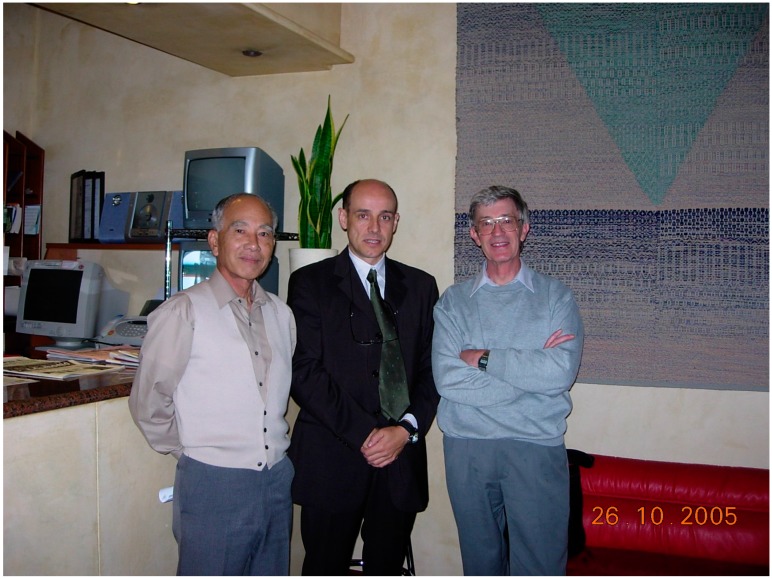
Photograph of Takeshi Yasumoto (left), Luis Botana (middle) and Rex Munday (right) in 2005.

